# Osteochondral regeneration with a novel aragonite-hyaluronate biphasic scaffold: up to 12-month follow-up study in a goat model

**DOI:** 10.1186/s13018-015-0211-y

**Published:** 2015-05-28

**Authors:** Elizaveta Kon, Giuseppe Filardo, Jonathan Shani, Nir Altschuler, Andrew Levy, Ken Zaslav, John E. Eisman, Dror Robinson

**Affiliations:** II Orthopedic division and NanoBiotechnology Lab, Rizzoli Orthopedic Institute, Bologna, Italy; Havat Daat Co., Beit Berl, Kfar Saba, Israel; Cartiheal Ltd., Atir Yeda 17, Kfar Saba, Israel; Center for Advanced Sports Medicine, Knee and Shoulder, Millburn, NJ USA; Cartilage Restoration Center: Advanced Orthopedic Centers and Clinical Prof. Orthopedic Surgery V.C.U. Med. Ctr., Richmond, VA USA; Osteoporosis and Translational Research, UNSW University, Sydney, NSW Australia; Department of Orthopedics, Rabin Medical Center, Petah Tikwa, Israel

**Keywords:** Osteochondral defect, Cartilage regeneration, Aragonite-hyaluronate, Agili-C

## Abstract

**Background:**

The regeneration of articular hyaline cartilage remains an elusive goal despite years of research. Recently, an aragonite-hyaluronate (Ar-HA) biphasic scaffold has been described capable of cartilage regeneration over a 6-month follow-up period. This study was conducted in order to assess the fate of the regenerated osteochondral tissue in a 12-month-long validated caprine model.

**Hypothesis/purpose:**

The hypothesis was that the implantation of the Ar-HA implant leads to tissue regeneration and maturation.

**Study design:**

A two-arm caprine model of a critical osteochondral defect compares the fate of acute osteochondral defects (group A) to Ar-HA implanted defects (group B).

**Methods:**

Critical 6 mm in diameter and 10-mm in depth osteochondral defects were created in the load-bearing medial femoral condyle of 20 mature goats and randomized into two groups. In group A (*n* = 6), a blood clot spontaneously filled the defect; in group B (*n* = 14), a single Ar-HA implant reconstructed the defect. The animals were sacrificed after either 6 or 12 months. Parameters assessed included clinical evaluation, x-rays, micro-CT, ultrasound and histology at both time points, and specimen high-field magnetic resonance imaging with T2 mapping at the 12-month time point.

**Results:**

In most group A animals, the defects were not reconstructed (1/3 at 6 months, and 0/3 at 12 months). Defects in group B were mostly reconstructed (5/7 at 6 months and 6/7 at 12 months). Group A defects were either empty or contained fibrous repair tissue; while group B filling was compatible with hyaline cartilage and normal bone.

**Conclusion:**

Ar-HA scaffolds implanted in critical osteochondral defects result in hyaline cartilage formation and subchondral bone regeneration. The results improved at the 12-month time point compared to the 6-month time point, indicating a continuous maturation process without deterioration of the repair tissue.

**Clinical relevance:**

Osteochondral defects are common in humans; the results of the current study suggest that an acellular Ar-HA scaffold might induce cartilage and subchondral bone regeneration.

## Introduction

Numerous surgical approaches have been proposed over the years to treat chondral or osteochondral lesions [1, 2], but native hyaline cartilage regeneration has not been achieved by any available treatment. In general, two approaches to treat articular cartilage are feasible: exogenous-cell-based techniques or locally recruited stem-cell-based techniques. Sophisticated cell-based technologies are feasible but difficult to justify in this economy-conscious age [3]. Regenerative scaffold-based procedures are emerging as a promising therapeutic option for the treatment of chondral lesions. Several one-step scaffold-based strategies are proposed to simplify the procedure and reduce costs [4, 5]. The properties of the graft can be specifically tailored to provide structural, biological and biomechanical cues that are necessary for a reproducible and durable repair. The optimal scaffold should allow bone repair in the subchondral area (a goal quite difficult to achieve as the bone tends to be a mixture of compact and woven bone [6]) and migration of mesenchymal stem cells and chondrocytes into the superficial cartilaginous layers, with new cartilage formation at the articular surface [6]. The regenerated cartilage should integrate into both the basal tissue as well as the surrounding peripheral cartilage [7, 8].

A recent study showed the potential of a newly developed aragonite-hyaluronate (Ar-HA) scaffold (Agili-C^TM^, CartiHeal (2009) Ltd., Israel) as an ideal composite material with biological and mechanical properties for chondral and osteochondral regeneration [9]. Safety and cartilage regeneration potential were evaluated in a goat model with 6-month follow-up. Kon et al. [9] described an optimal coralline aragonite scaffold configuration—with a drilled channel pattern impregnated with hyaluronic acid (HA) at the articular surface. The cartilaginous repair tissue presented a smooth contour and was well integrated into the adjacent native cartilage, with morphological evidence of hyaline cartilage, confirmed by the presence of proteoglycans and collagen type II and the absence of collagen type I. Initial human clinical experience with the Ar-HA scaffold was obtained when implanted in an Outerbridge grade IV medial condylar defect measuring about 2 cm^2^. Six months following the procedure, the patient returned to sport activities and a 24-month MRI confirmed articular surface restoration [10].

A spectre haunting cartilage repair therapies is the long-term outcome. The purpose of this study was to confirm the durability of the regenerated hyaline cartilage in a caprine model over a 1-year period. The study hypothesis was that the modified Ar-HA based implant leads to the regeneration of long-term durable hyaline cartilage.

## Materials and methods

### Implanted device

**Fig. 1:**
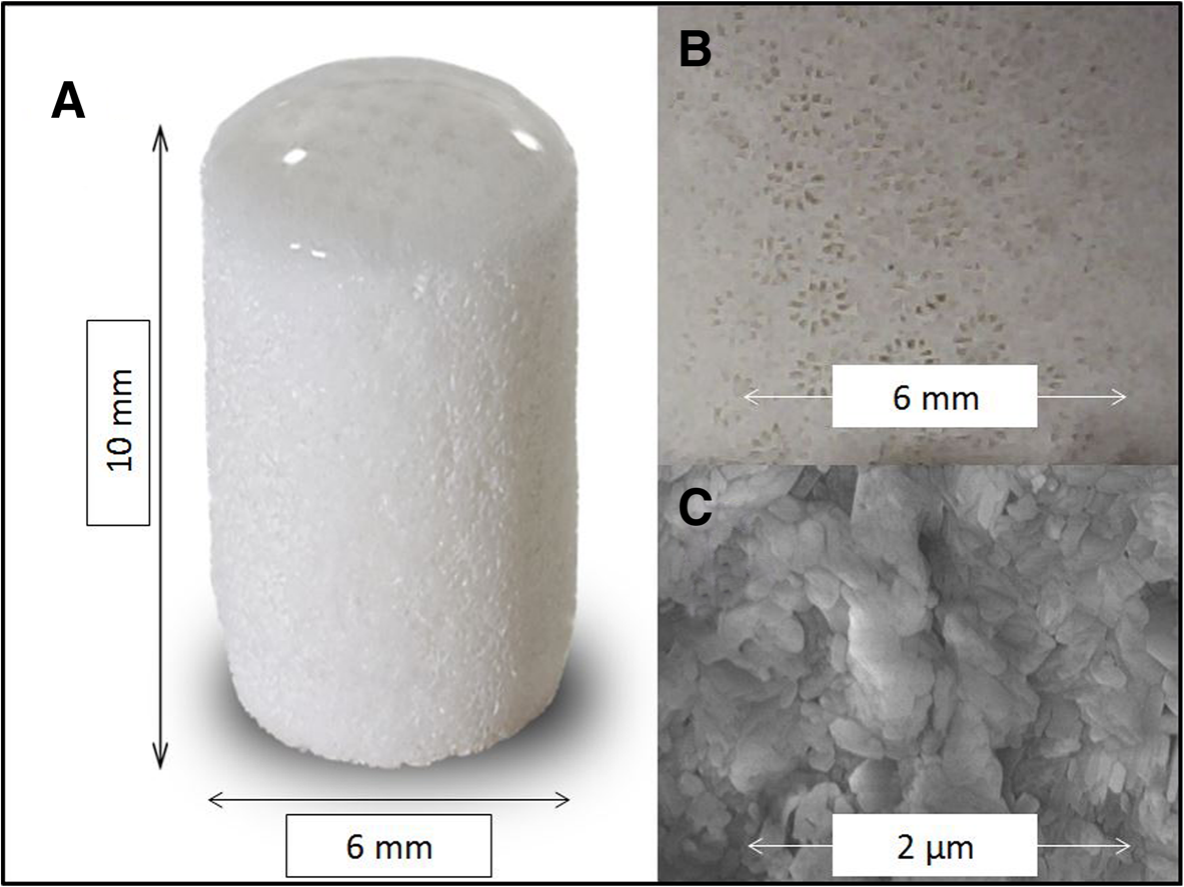
Micrograph of the Ar-HA scaffold (**a**), the biphasic nature is obvious with a thin hyaluronate covered cartilage phase, overlying a thick bone phase. Close-up of the bone phase demonstrates the typical corallites structure with radial septo-costae (**b**). The coral material is nano-rough making it a stem cell attractor (**c**)

**Fig. 2:**
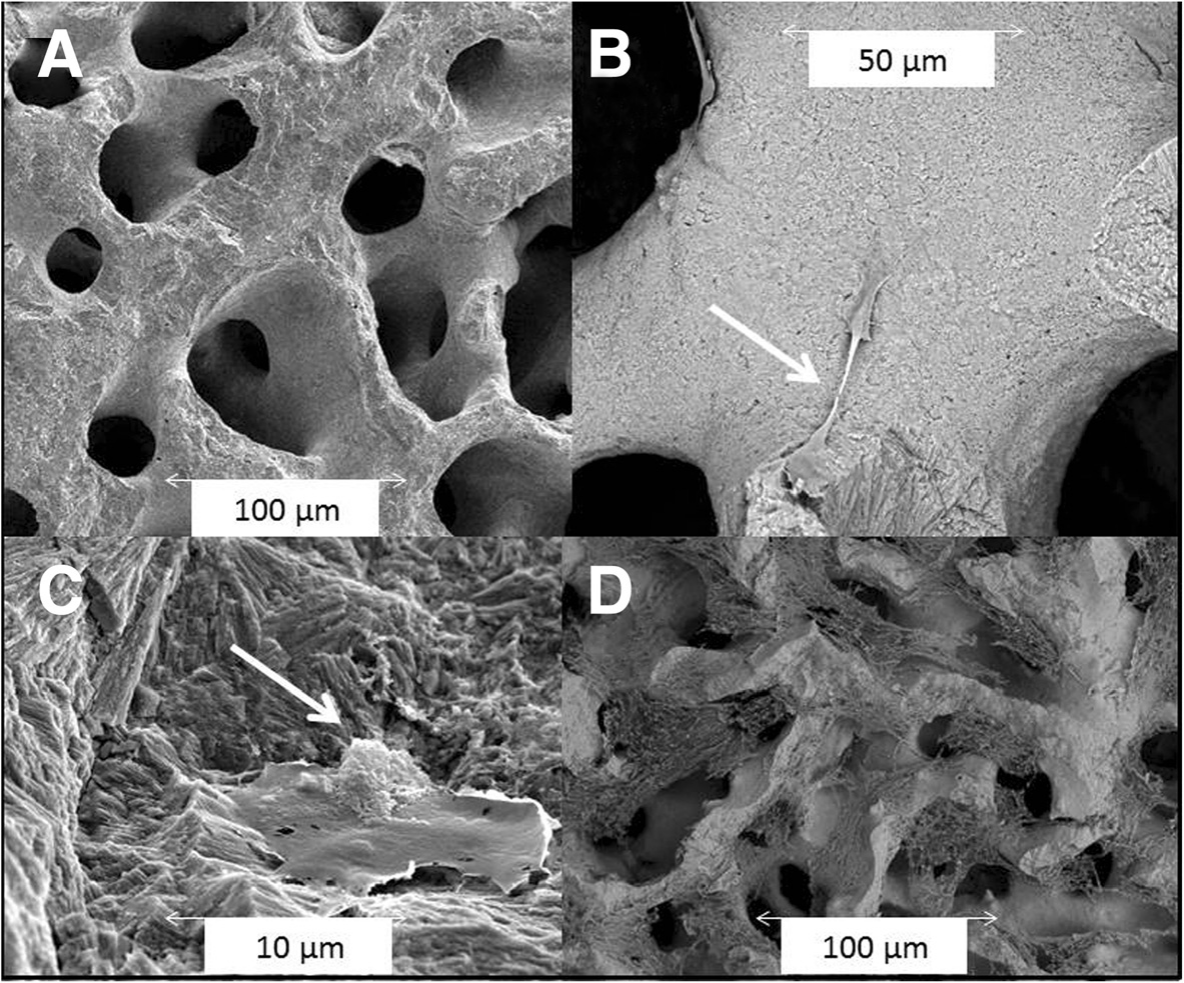
The Ar-HA scaffold has an interconnecting porosity, with an average of 100-μm pore size (**a**). The coral material supports human stem cell adhesion and mitosis (*arrow*) as soon as 24 h after seeding (**b**). A larger magnification demonstrates that 24 h after seeding the human stem cells are well attached to the nano-rough surface and deposit a substance on the side opposite to the scaffold (presumably osteoid, *arrow*) (**c**). Seven days after seeding, tissue is covering the scaffold and partially filling the pores (**d**) (environmental scanning electron microscopy of human embryonic palatal mesenchyme (HEPM) cells, seeded at about 15,000 cells per well, were seeded in a 48-well plate on scaffolds. Cells were grown in HEPM growth medium for up to 7 days)

The implanted device has been previously described both in animal studies and in human implantation [9, 10]. It is an Ar-HA biphasic implant (Fig. 1). Its unique nano-rough structure and the interconnecting porosity stimulate cell adhesion and proliferation as well as matrix production (Fig. 2).

### Experimental model

**Fig. 3:**
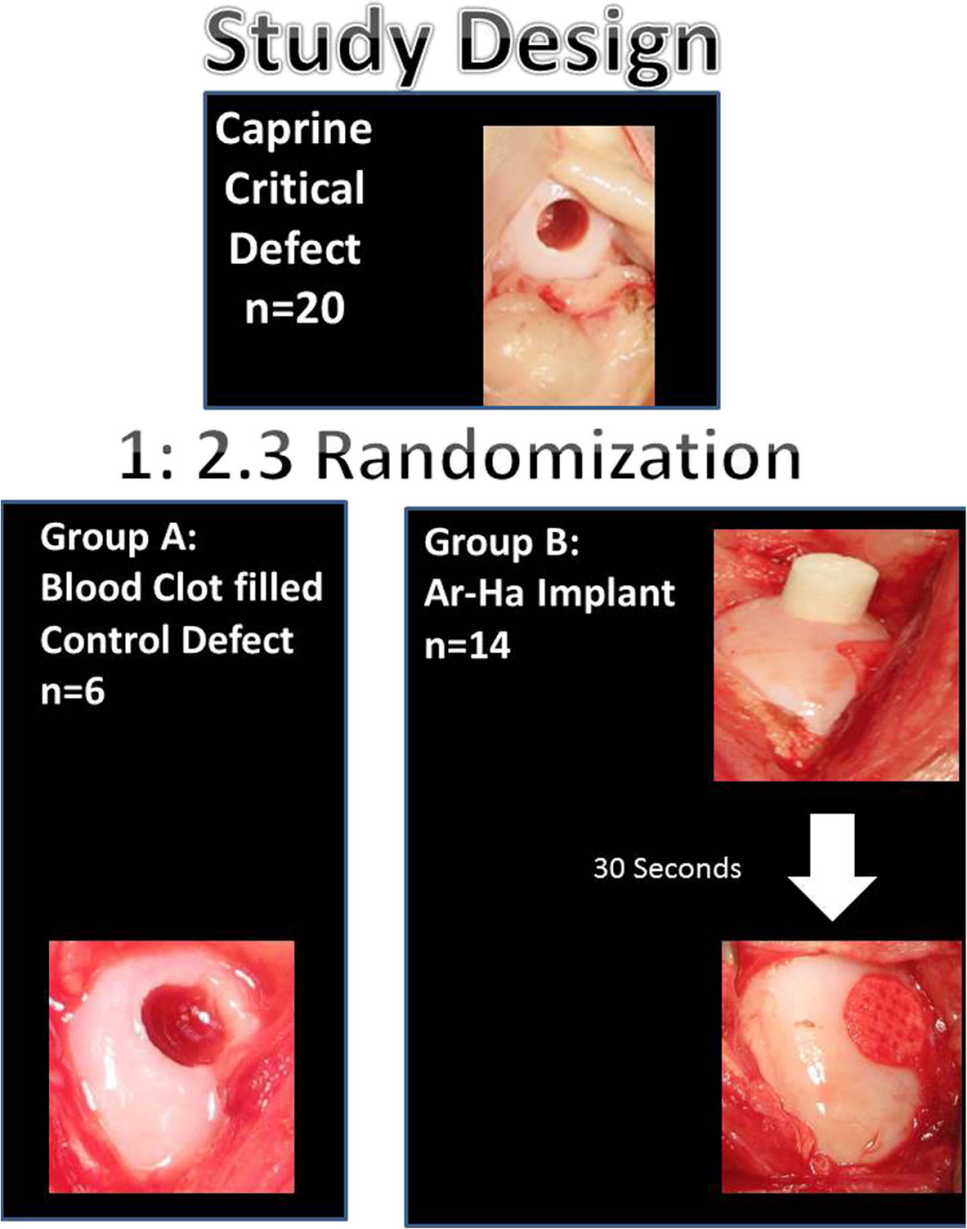
The study included 20 animals in whose right medial femoral condyle, a critical size defect was created. The animals were randomized at 1:2.3 ratio into two groups: group A—the defect was created and blood was allowed to clot in it; this group served as a control group, group B—experimental group—Ar-HA-implanted group

One of the recommended experimental models for osteochondral defect evaluation is a goat model [11]. Goats and sheep are readily available from commercial and agricultural suppliers as 2-year-old or older animals that are skeletally mature. According to ASTM F2451 largely due to their stifle size, cartilaginous thickness, availability, and ease of handling, goats represent a favorable animal model for cartilage repair studies [12]. The lesion size is important, and critical-size defects should be used for pivotal studies. In such pre-clinical study, the choice of comparator is important. A 7-mm diameter defect has been shown to lead to osteoarthritic changes in an ovine model [13]. Thus, a similar defect (slightly smaller due to slightly smaller caprine size) was chosen as a critical defect in our study based on previous studies demonstrating lack of spontaneous repair at this defect diameter[14, 9, 13]. The overall study design is delineated in Fig. 3. As the model of blood-clot-filled defect is well known for its lack of healing ability in the goat [15, 13], a 1:2.3 randomization scheme was used.

### Rationale for choice of control group

The current study aimed at assessing the repair augmentation of acute osteochondral defects with an acellular scaffold as compared with blood clot. Results with chronic defects have been shown to be similar previously with clear superiority of osteochondral scaffolds filling the defects as compared with spontaneous healing [14]. Clots are known to allow some tissue regeneration; however, regarding the goat model, it is well known that full-thickness osteochondral defects, measuring 6 mm in both diameter and depth, that are created in the medial femoral condyle of the knee joint of adult Spanish goats do not heal [15]. Clot-filled empty defect seems to be an appropriate control group choice as it simulates the occurrence following trauma in humans and the repair reaction generated. It is well known that even joints that appear to self-heal 6 months after osteochondral defect creation, later undergo degeneration at 12 months post-defect creation [13, 16]. Due to these observations and as there is no gold-standard treatment of osteochondral defects in current clinical practice, an empty osteochondral defect was chosen as a comparator in the current study in line with ICRS guidelines [11] and the FDA draft guidance [12, 17].

### Animals

Twenty skeletally mature, female, Saanen goats, 48 ± 6 kg average weight (41–61 kg), non-pregnant and non-lactating, were acquired from an authorized farm and quarantined for at least 45 days before entering the trial. The goats used in this study were screened for caprine encephalitis prior to inclusion in the cohort group as it is a common cause of adult goat arthritis.

Six animals served as a control group (group A), with an empty defect 6 mm in diameter and 10-mm depth that spontaneously filled with blood clot within a few minutes (Fig. 3) created in the medial load bearing area of the MFC of the right hind-limb. Fourteen animals were implanted with a single implant (group B), inserted into an osteochondral defect created in the middle part of the load-bearing medial femoral condyle (MFC) of the right hind-limb (Fig. 1b).

Animal care and surgery were approved by the Ethical Committee of the Experimental Center and performed under the Animal Welfare Law and according to applicable ISO 10993 Standards. The study was conducted in accordance with the following standards:

ASTM F2451-05 Standard Guide for in vivo Assessment of Implantable Devices Intended to Repair or Regenerate Articular Cartilage.

Preclinical Studies for Cartilage Repair: Recommendation from the International Cartilage Repair Society; ICRS Recommendation Papers, Cartilage 2(2) 137–152, 2011.

International Cartilage Repair Society (ICRS) Recommended Guidelines for Histological Endpoints for Cartilage Repair Studies in Animal Models and Clinical Trials, Cartilage 2 (2) 153–172, 2011.

Three group A animals and seven group B animals were sacrificed at each timepoint. The study was approved by the Assaf Harofe Medical Center Animal Care Committee (Zeriffin, Israel).

### Surgical procedure

Surgery was conducted under general anesthesia via a mini-arthrotomy approach. A minimal exposure of the implantation site was made using retractors with the limb placed at maximal flexion, allowing joint access without patellar dislocation. A specifically developed surgical tool set, (Agili-Kit^TM^, CartiHeal (2009) Ltd., Israel) was used for the implantation. After defect creation an implant was inserted. The implant reached its final position in a press-fit manner, slightly below the articular surface. The knee capsule and skin were then sutured.

Intramuscular antibiotics (Cephalexin, 1 g ×3 for 24 h) and oral analgesics (Dipyrone, P.O. 1 g per day for 24 h) were administrated postoperatively. Following the surgical procedure, the goats were placed in protected stalls with limited space for movement (2 × 2 m) with immediate load-bearing and *ad libitum* food and water supply. After 10 days the goats were transferred to a study specific research pen allowing unhindered ambulation. The goats’ welfare was monitored by a veterinarian on a routine basis throughout the study. Ten animals were evaluated 6 months post implantation and the other ten animals were evaluated 12 months post implantation. Animals were euthanized by pharmacological premedication (ketamine (3 mg/kg), xylazine (0.1 mg/kg) and an injection of 50 cc KCl (150 mg/ml). Prior to euthanasia, clinical evaluation, blood tests and ultrasonography imaging were performed. After sacrifice, macroscopic and histological analysis as well as x-rays, micro-CT, and MRI of the explanted specimens were performed in a blinded manner.

### Repair tissue evaluation

#### Ultrasound

Ultrasound imaging of the operated joint was performed prior to sacrifice, as the procedure requires physical subduing of the tested animal and to prevent the possible interference effect from this physical manipulation with other assessments the examination was performed 5 months post procedure, during the in-vivo phase, for the entire animal group (about 3 weeks prior to sacrifice of some of the animals). An additional ultrasound was performed 11 months post procedure (again in order to allow enough time to elapse prior to sacrifice) for the 12 month group. The ultrasound transducer used was GE Transducer 11L Logiq_e® 12 MHz. Evaluation of the ultrasound results according to gross appearance scaling system modified from Fortier et. al [18] and ICRS macroscopic cartilage assessment score [19] was performed in a blinded manner by an independent veterinarian radiologist.

### X-rays

X-rays were performed on the right and left hind-limbs of the goats, i.e., the operated and non-operated knees for comparison. The radiographic imaging was conducted after joint harvesting and before macroscopic evaluations, using a GE OEC-9800 C-arm system. The radiographs were evaluated in a blinded manner for the following parameters: bone cysts, osteophytes, subchondral sclerosis, joint space narrowing and other arthritic changes.

### Macroscopic evaluation

At both time points (6 and 12 month’s follow-up), macroscopic evaluation was conducted in a blinded manner by a team of human and veterinary orthopedic surgeons. The evaluations performed were: ICRS macroscopic cartilage assessment scoring [19] and gross appearance scaling system (modified from Fortier et al.) [18]. The opposing articular surface was also evaluated.

### Micro-CT

Micro CT was performed at the 12-month time point on the harvested condyles, using a TomoScope® Synergy stand-alone in-vivo micro-CT scanner (scan time 90 s, one gantry rotation, radiation dose 322 mGy/cm, tube voltage 65 kV, current 1 mAmp). This dedicated small animal CT system has an 80 μm resolution. Bone density was calculated as occupied bone trabeculae area divided by total region of interest area. Structural density was calculated at both the repair site and the periphery. Each value represents averaging of three randomly chosen ×20 magnification fields. The density at the periphery was assumed to represent bone affected indirectly by the defect creation and somewhat similar to normal bone. Thus, subtraction of repair site structural density from the peripheral bone density allows evaluation of the procedure specific bone formation.

### MRI imaging and data analysis

At the 12-month time point, the specimens were scanned in a 7T MRI system (Bruker, Germany). A T2 protocol was performed (T2-MSME sequence; TR = 1844.32 ms; ten different TEs: 8; 16; 24; 32; 40; 48; 56; 64; 72; 80 ms; number of averages: 3; scan time: 11 min 48 s 218 ms; matrix: 160 × 160 pixel; spatial resolution: 0.15625 mm/pixel × 0.15625 mm/pixel; slice thickness: 0.8 mm). The images produced allowed assessment of the cartilage (appearing as a high signal tissue due to its high water content, and the subchondral bone that appears as a very low signal tissue). With quantitative T2 mapping, the collagen structure and water content can be appreciated [20]. A quantitative T2 map was generated for each sample from the T2 weighted images using in-house software written in MATLAB® (MathWorks, Natick, MA, USA). T2 relaxation time non-linear fit in each voxel was calculated according to the equation *S*(*t*) = *S*(0)exp(−*t*/T2)/2/. The T2 images were presented in a color coded map representing the T2 values. Regions of interest (ROIs) were collected from two areas: newly formed cartilage at the implantation site and peripheral cartilage proximal to the implantation site.

Additionally, cartilage thickness was measured using ImageJ 1.45S software by NIH USA (Wayne Rasband), by a “straight line” measurement tool at four random points in each defect and in the peripheral area of the scan. Specimens were scored blinded according to the New 3-D MOCART score [21] by an independent veterinarian radiologist (modified to exclude the effusion parameter as isolated specimens were evaluated and effusion could not be evaluated).

### Histology and immunohistochemistry

Gross specimens were sent to an independent laboratory, NAMSA—BIOMATECH (Zl De L’Islon 115 Rue Pasteur, France) for histological preparations and evaluations at both sacrifice time points. The specimens, embedded in paraffin blocks, were longitudinally cut (5 μm thickness +/−0.5 μm) using a microtome (MICROM®, France). For each specimen, six central serial sections were prepared. Safranin hematoxylin eosin (SHE) staining was used for analysis of the inflammatory reaction; Masson’s trichrome (MT) for general morphology assessment including analysis of the fibrous tissue pattern; Safranin-O/Fast Green (SOFG) histochemical staining for the proteoglycan content (the SOFG staining was simultaneously carried out in the same bath to avoid stain variability); Feulgen and Rossenbeck histochemical staining for quantitative analysis of the cartilage cellularity. Other two sections were used for the immunohistochemistry (IHC) determination of collagen type I (Coll I) (fibrous tissue marker, polyclonal antibody obtained from Abcam plc., 330 Cambridge Science Park, Cambridge, UK, Ref. ab90395 at dilution 1:100) and collagen type II (Coll II) (hyaline cartilage marker, polyclonal antibody obtained from Abcam, Ref. ab34712 at dilution 1:800). Slides were then incubated with a biotylinated link (Dako France S.A.S. Parc Technopolis, 3 Avenue du Canada, 91978 Les Ulis Cedex; Kit reference K5001) for 15 min at room temperature. All slides were visualized with 3,3-diaminobenzidine chromogen + (DAKO; Kit reference K5001) and a Mayer hematoxylin counterstain. Semi-quantitative evaluation of the local tissue effects was performed to assess the inflammatory reaction (polymorphonuclear cells, lymphocytes, plasma cells, macrophages and giant cells/osteoclasts), fibrin, necrosis, osteolysis and neovessels using a 0–4 grading scale. The performance parameters were graded according to a combination of the ICRS II grading scale and the O’Driscoll et al. grading scale [22] (see Table 1); the score was described as a total performance score (TPS) ranging from 0–36 points according to the following parameters: nature of predominant tissue (tissue morphology and Safranin-O/Fast Green staining of the matrix), structural characteristics (surface regularity, structural integrity, thickness, bonding to the adjacent cartilage, basal integration, tidemark, subchondral bone abnormalities/marrow fibrosis, cancellous bone regeneration and abnormal calcification/ossification), freedom from cellular changes of degeneration (hypocellularity and chondrocytes clustering), freedom from degenerative changes in adjacent cartilage and overall assessment. Slides were evaluated under polarized light for the determination of the collagen organization. The immune-labeled slides were qualitatively evaluated for the presence of collagen type I and type II. Alickert-type 5 point semi-quantitative score ranging from 0 to 4 was used to score overall assessment (OA).

**Table 1:** Combined cartilage and bone repair score evaluation (from ICRS II-2010 and O’Driscoll et al.)

Nature of predominant tissue		Structural characteristics									Freedom from cellular changes of degeneration		Freedom from degenerative changes in adjacent cartilage	Immunohistochemistry labeling	Overall assessment
Tissue morphology (including use of polarized light)	Safranin-O staining of the matrix	Surface regularity	Structural integrity	Thickness	Bonding to the adjacent cartilage	Basal integration	Formation of a tidemark	Subchondral bone abnormalities/marrow fibrosis	Cancellous bone regeneration	Abnormal calcification/ossification (cartilage tissue)	Hypocellularity	Chondrocyte clustering			
Fibrous tissue or bone : 0	None: 0	Severe disruption, including fibrillation: 0	Severe disintegration: 0	0–50 % of normal cartilage: 0	Not bonded: 0	No basal integration: 0	Absence of visible interface: 0	Irregular subchondral plate, fracture, sclerosis, intense remodeling: 0	Slight: 1	No ectopic change: 0	Severe hypocellularity: 0	25–100 % of the cells: 0	Severe hypocellularity, poor or no staining: 0	-: No labeling	Fibrous tissue: 0
Incompletely differentiated mesenchyme: 2	Slight: 1	Fissures 25–100 % of the thickness : 1	Slight disruption, including cysts: 1	50–100 % of normal cartilage: 1	Bonded at one end, or partially at both ends: 1	Partial integration: 2	Slight to moderate calcification front/tidemark: 2	Absence of abnormality: 3	Moderate (with residual fibrous tissue/cyst): 2	Presence of changes: 3	Moderate hypocellularity: 1	<25 % of the cells: 1	Mild or moderate hypocellularity, slight staining: 1	1: Slight labeling	Fibrocartilage tissue: 2
Hyaline articular cartilage: 4	Moderate: 2	Superficial horizontal lamination : 2	Normal: 2	100 % of normal adjacent cartilage: 2	Bonded at both ends of graft : 2	Full integration: 4	A single tidemark (signs of maturation): 3	/	Marked (limited residual fibrocartilage tissue): 3	/	Slight hypocellularity: 2	No clusters: 2	Normal cellularity, mild clusters, moderate staining: 2	2: Moderate labeling	Articular and hyaline cartilage tissue: 4
/	Normal or nearly normal: 3	Smooth and intact: 3	/	/	/	/	/	/	Complete: 4	/	Normal cellularity: 3	/	Normal cellularity, no clusters, normal staining: 3	3: Marked labeling	/

The histological preparation and evaluation was conducted in compliance with the OCDE Good Laboratory Practice, ENV/MC/CHEM (98) 17, section II § 2.2 and 9.2, with the European Good Laboratory Practice regulations, 2004/10/EC Directive, section II § 2.2 and 9.2 and with the United States Food and Drug Administration Good Laboratory Practice regulations, 21 CFR 58 subparts B 35 (b) and J 185 (a). All evaluations were performed in a blinded fashion by a veterinary pathologist (NAMSA).

### Statistical method

Statistical analyses were performed using Analyse-It statistical Microsoft 2010 Excel add-in (version 3.70.1). A *p*-value of 0.05 or less was considered statistically significant. Continuous variables with normal distribution were compared using the Student’s *t*-test or Welch ANOVA. Non-parametric variables were analyzed using the Wilcoxon–Mann–Whitney test or the Kruskal-Wallis test as appropriate.

## Results

All goats recovered well and quickly returned to normal walking ability within 2 days of surgery. In the study animals, there was no evidence of any movement restriction or joint locking in any of the cases. One (SAE) serious adverse event occurred during the study; a group A animal suffered from cysticercosis (considered to be unrelated to the study). This led to death 5 days before scheduled sacrifice (the joint was retrieved for analysis according to the protocol). Minor adverse events occurred similarly (weight loss, high temperature, sneezing) in both groups. Group A animals gained 5 ± 15 % of total body weight while group B animals gained an average of 13 ± 15 % of total body weight (Student’s *t*-test, *t*-statistic = −.091, difference not significant [n.s.]).

### Ultrasound

The gross appearance scaling system score and ICRS US score were significantly superior in group B at both time points, as detailed in Tables 2 and 3. While ultrasound is not commonly used in cartilage repair studies, it has been shown to allow assessment of cartilage thickness in the human knee and has shown a close correlation with symptoms [23].

**Table 2:** Gross appearance scaling system

Fortier gross appearance scaling system	6 months*	12 months**
Min = 0, max = 15		
Ar-HA-implanted group	9.71 ± 3.01 (*n* = 14)	13.71 ± 0.88 (*n* = 7)
Control group	2.66 ± 3.59 (*n* = 6)	5.33 ± 3.29 (*n* = 3)

*Wilcoxon–Mann–Whitney test, *w*-statistic 29, *p* < 0.004

**Wilcoxon–Mann–Whitney test, *w*-statistic 6, *p* < 0.002

**Table 3:** ICRS US score

ICRS US evaluation Min = 0, max = 12	6 months*	12 months**
Ar-HA-implanted group	10.07 ± 1.63 (*n* = 14)	11.85 ± 0.34 (*n* = 7)
Control group	2.66 ± 3.14 (*n* = 6)	5.66 ± 3.39 (*n* = 3)

*Wilcoxon–Mann–Whitney test, *w*-statistic 25, *p* < 0.001

**Wilcoxon–Mann–Whitney test, *w*-statistic 6, *p* < 0.002

### Macroscopic evaluation

**Fig. 4:**
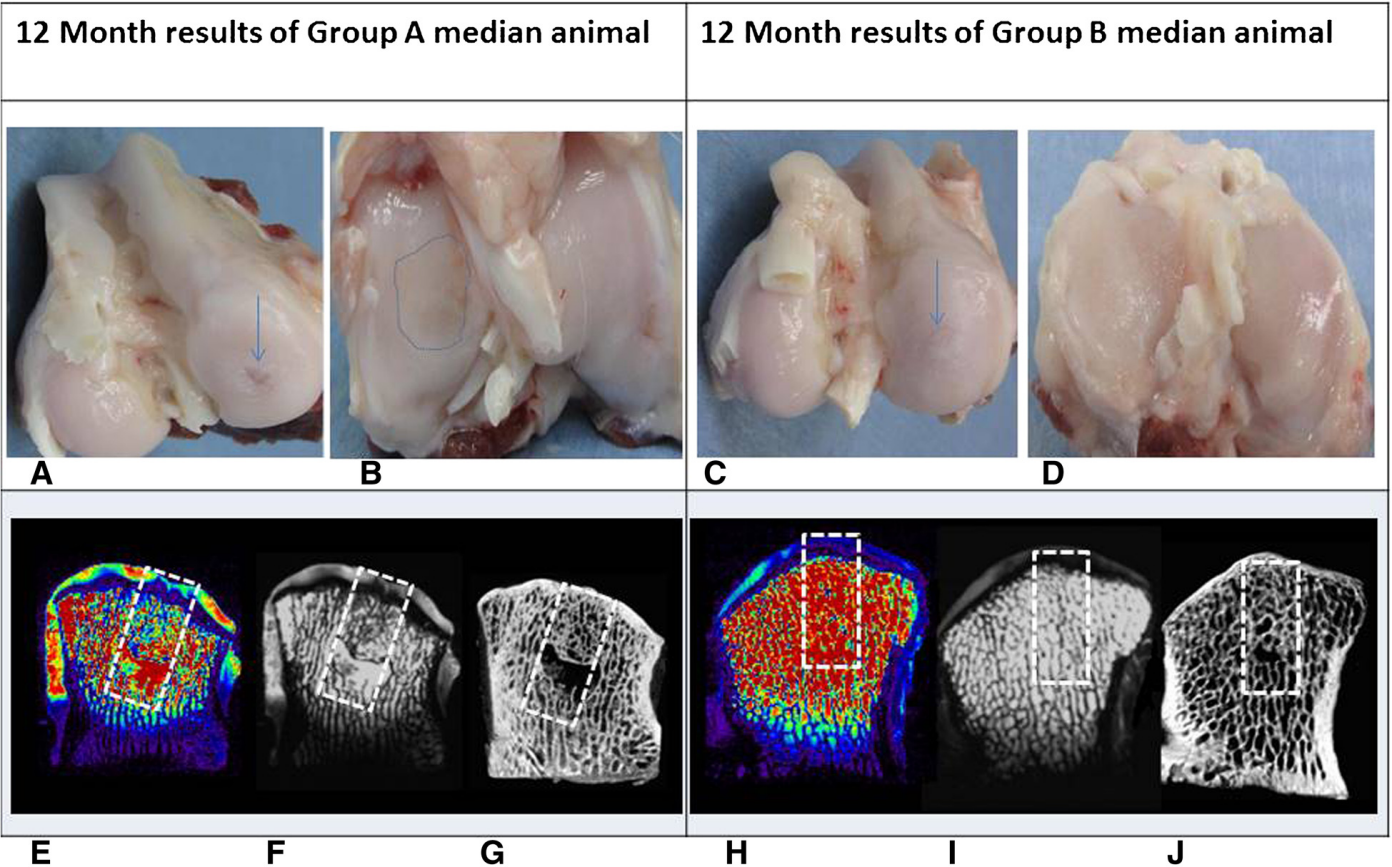
Results at 12 months post implantation of the median animal from group A (*left column*) and the median animal from group B (*right column*) using several evaluation modalities: **a** Macroscopic appearance of group A defect; a crater is visible at the site of the osteochondral defect (*arrow*). **b** Opposing articular surface of the tibia of same animal as (a) demonstrates a large area of tibial cartilage damage (dashed line). **c** Group B animal condyle appears to be perfectly covered with cartilage. **d** Opposing tibial cartilage in a group B animal is intact. **e** T2 mapping of a group A animal demonstrates abnormal signal indicating cartilage degeneration both overlying the defect (dashed box) and in the surrounding tissue. **f** T2 MRI scan demonstrates a cyst in the subchondral bone and abnormal dark signal in the superficial repair tissue supporting a fibrous content of the tissue. **g** A CT scan demonstrates some bone formation within the defect surrounding a large cyst and a lack of subchondral bone plate reconstruction. **h** Group B median animal demonstrates normal T2 mapping both within the tissue overlying the implant and in the periphery. Note the zonation of the repair tissue (blue-hued tissue overlying a purplish zone that is attached to the bone) indicating a likely hyaline cartilage nature of the repair tissue. **i** T2 scan of a group B animal indicates excellent subchondral bone regeneration with superficial tissue formation whose signal is typical of hyaline cartilage. **j** On CT scan, the bone tissue is fully reconstructed and the subchondral bone plate is similar in thickness and contour to its surroundings

The ICRS macroscopic cartilage assessment score was higher for group B compared to group A at both time-points (Fig. 4, n.s. difference at 6 months, and statistically significant at 12 months Wilcoxon–Mann–Whitney test, w-statistic 6, *p* < 0.02; Table 4). The Fortier’s gross appearance scaling system was significantly superior for group B at both time points (at 6 months, Wilcoxon–Mann–Whitney test, w-statistic 29, p < 0.004; at 12 months Wilcoxon–Mann–Whitney test, *w*-statistic 6, *p* < 0.02; Table 5).

**Table 4:** ICRS macroscopic cartilage assessment score

ICRS macroscopic evaluation	6 months	12 months
Min = 0, max = 12		
Ar-HA-implanted group	9.57 ± 1.39 (*n* = 7)	11.42 ± 0.72 (*n* = 7)
Control group	6.33 ± 4.49 (*n* = 3)	7.33 ± 0.47 (*n* = 3)

**Table 5:** Fortier’s gross appearance scaling system

Fortier gross appearance scaling system	6 months	12 months
Min = 0, max = 15		
Ar-HA-implanted group	12.71 ± 2.60 (*n* = 7)	13.71 ± 1.03 (*n* = 7)
Control group	5.66 ± 2.49 (*n* = 3)	9.33 ± 1.24 (*n* = 3)

No adverse reaction or fibrillation was noted at the opposing articular surface in either group.

### Micro-CT

Bone structural changes and anomalies, including cyst formation, were seen in group A while in group B animals, the bone structure was similar to normal bone.

At the 6-month time point, group B animals had structural density at the subchondral area of 56 ± 4 % and at the 12-month time point of 45 ± 3 %, which was similar to the peripheral area bone density (45 ± 2 %). Scaffold residuals, yet not fully degraded, explain the higher bone density at the 6-month time point versus the 12-month time point in group B. In group A, lower bone density values were measured at both 6 months (35 ± 3 %) and at the 12-month time point (34 ± 4 %). Due to the limited number of specimens, a statistical analysis was performed of the pooled data from both time points. The intergroup difference in structural density was found to be significant (ANOVA, *F*-statistic 4.94, *p* < 0.04).

### MRI imaging

Cartilage thickness-defect fill:

Six out of the 7 group B animals had cartilage which was similar in thickness to the surrounding cartilage. In one animal, regenerated cartilage thickness was thinner than the surrounding cartilage. By contrast, in all of the group A animals, tissue coverage over the defect area was thinner compared to the surrounding native cartilage (Fig. 4).

New MOCART scoring:

The 3D New MOCART evaluation showed a significant intergroup difference in favor of group B (Student’s *t*-test, *p* < 0.05) for the following parameters: defect fill, cartilage interface, bone interface, surface, structure, chondral intralesional osteophytes, bone marrow edema, and subchondral bone. The overall New MOCART score (scored as a sum of all parameters assessed) was also found to be significantly superior in group B animals (32 ± 1 versus 17 ± 1, *p* < 0.001; Table 6).

**Table 6:** MOCART score of specimens removed at animal sacrifice 12 months after operation

New 3D-MOCART score parameter	HA-AR group	Control group
	Mean ± SD	Mean ± SD
Defect fill	4 ± 0.1	2 ± 0.8
Cartilage interface	4 ± 0.1	2 ± 0.8
Bone interface	4 ± 0.1	3 ± 0.8
Surface	3.9 ± 0.3	2.3 ± 0.5
Structure	1.9 ± 0.3	1 ± 0.1
Signal intensity	2.4 ± 0.5	1 ± 0.1
Subchondral lamina	1.9 ± 0.3	1.3 ± 0.5
Chondral osteophytes	2.6 ± 0.7	1 ± 0.1
Bone marrow edema	4 ± 0.1	2.7 ± 0.9
Subchondral bone	3.4 ± 0.9	1.7 ± 0.5
Overall score	32 ± 1	17 ± 1

At the 12-month time point, significantly lower (Student’s *t*-test, *p* < 0.001) T2 values were found in group B in the regenerated tissue overlying the defect (22.3 ± 9.6) versus group A (4.93 ± 9.9), which implies better quality of cartilage formation. Twelve months following creation of an osteochondral defect in the group A animals, there was also evidence of surrounding cartilage deterioration with significantly higher T2 values in group A animals (42.9 ± 29.9) as compared to the T2 values found at the peripheral cartilage of group B animals (27.0 ± 9.3, Student’s *t*-test, *p* < 0.05).

### Histology

#### Summary

**Fig. 5:**
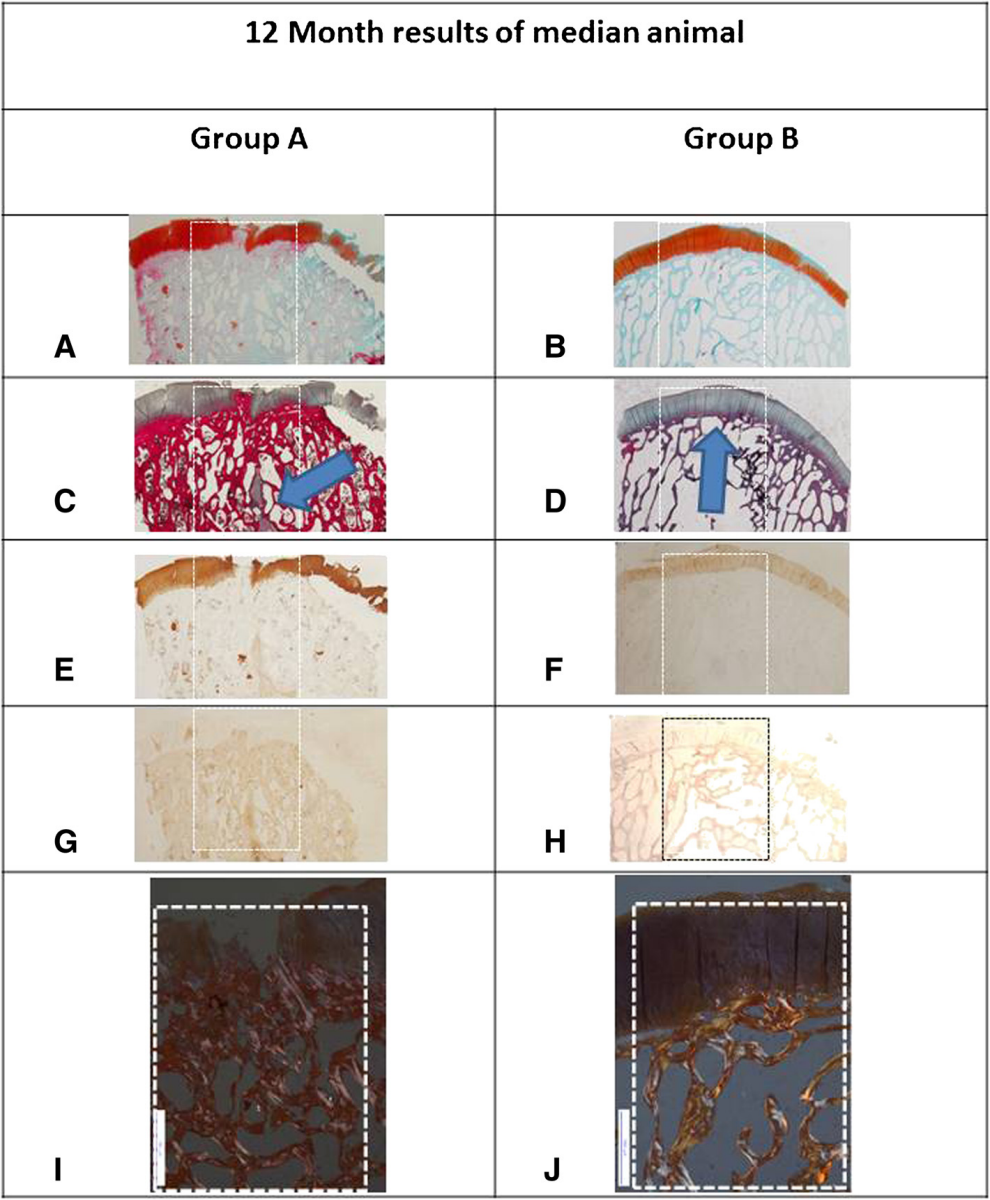
Histological evaluation of osteochondral defects 12 months postop (original magnification of (a–h) ×12, white dashed box is the osteochondral defect outline). **a** A proteoglycan-specific dye stains the cartilage layer in a group A animal. The osteochondral defect is still not reconstructed with eburnated bone at the joint surface. Note cartilage disruption, cleft formation, cartilage matrix loss. disrupted subchondral bone plate, and irregular bone trabeculae (Safranin-O stain, Fast Green counterstain). **b** A group B animal showing normal cartilage. **c** A bone cyst is seen in a group A animal (*arrowhead*), with massive thickening of the subchondral bone plate (Masson Trichrome stain). **d** A group B animal demonstrates perfect repair of the subchondral bone underlying the regenerated cartilage (*arrowhead*) (Masson Trichrome stain). **e** Collagen type II staining reveals lack of collagen type II in some of the arthritic tissue overlying the osteochondral defect with some staining in the bone cyst indicating heterotopic cartilage formation in a group A animal (anti-collagen type I antibody). **f** Collagen type II stain is uniform in the newly created cartilage in a group B animal; there is no collagen type II staining in the regenerated subchondral bone (anti-collagen type II antibody). **g** Collagen type I stain is uniform in the bone, but there are areas within the articular cartilage that stain positively as well in a group A animal (anti-collagen type I antibody). **h** Uniform collagen type I stain in a group B animal (anti-collagen type I antibody). **i** Disordered repair collagen fibrils are evident in this polarized light microscopy image of a group A animal and lack of repair tissue zonation (original magnification of this and **j** was ×40). (j) Well-ordered mature collagen fibrils are evident within the subchondral bone in a group B animal. The cartilage layer demonstrates a clear zonation phenomenon

After 6 and 12 months, no significant local inflammation was observed in both groups (see Table 7 and Fig. 5). At 6 months, most group B specimens (5/7) were associated with a high level of healing performance, with formation of young articular hyaline cartilage and advanced signs of subchondral bone regeneration, resulting in an average total performance score (TPS) of 31.0 ± 4.5 in group B as compared to group A with a TPS of 16.3 ± 14.5 (ANOVA test, *F*-value 6.68, *p* < 0.035). The large variability of the group A repair reaction is related to the fact that in two animals, the defects were not healed but in one animal, the defect healed spontaneously with irregular subchondral bone.

**Table 7:** Semi-quantitative histopathological analysis of the inflammatory reaction (results)

Time period	Group		Polymorphonuclear cells	Lymphocytes	Plasma cells	Macrophages	Giant cells/osteoclasts	Fibrin	Necrosis	Osteolysis	Neovessels
6 months	Agili-C	Mean	0.0	0.6	0.0	1.3	1.0	0.9	0.0	0.0	1.6
		*SD*	*0.0*	*0.5*	*0.0*	*0.5*	*0.0*	*0.8*	*0.0*	*0.0*	*0.5*
	Empty defect control	Mean	0.0	0.0	0.0	0.3	1.0	1.0	0.0	0.0	1.7
		*SD*	*0.0*	*0.0*	*0.0*	*0.5*	*0.0*	*0.8*	*0.0*	*0.0*	*0.5*
12 months	Agili-C	Mean	0.0	0.3	0.0	0.7	1.1	0.1	0.0	0.3	0.0
		*SD*	*0.0*	*0.7*	*0.0*	*0.9*	*0.3*	*0.3*	*0.0*	*0.7*	*0.0*
	Empty defect control	Mean	0.0	0.3	0.0	0.7	1.3	0.3	0.0	0.0	1.0
		*SD*	*0.0*	*0.5*	*0.0*	*0.5*	*0.5*	*0.5*	*0.0*	*0.0*	*0.8*

Grading scale: 0: absence, 1: slight, 2: moderate, 3: marked, 4: severe SD: standard deviation

At 12 months, the group B healing features (TPS) were significantly higher than group A’s TPS (ANOVA test, *F*-value 9.09, *p* < 0.02) with 6/7 healed specimens with hyaline cartilage tissue and subchondral bone and the seventh specimen healed with almost complete hyaline cartilage tissue and high TPS (33.0 ± 5.9). In contrast, none of the group A specimens were fully repaired and the TPS was low (18.0 ± 7.2). Comparing the group A TPS score at both time points demonstrate that the healing was similar over time (ANOVA Dunnet multiple comparisons against control, mean difference 1.7, n.s.). Between 6 and 12 months, the group B’s TPS further improved, albeit the difference was not statistically significant.

The overall assessment at 6 months was significantly lower (2 ± 1.6) for the group A versus the group B animals (3.43 ± 0.9, Kruskal-Wallis test, *H*-statistic 9.85, *p* < 0.02).

In order to evaluate the collagen type II content of the repair tissue, the data were pooled for the 6-months and 12-month group due to the few animals in the control group. Group A had significantly less collagen type II staining (median 2.5, range 0–3) versus group B (median 3, range 2–3, Student’s *t*-test, *t*-statistic −2.7, *p* < 0.015). Safranin-O staining, indicating proteoglycan content of the repair tissue was higher in group B (2.6 ± 0.6) versus group A (2.2 ± 1, Student’s *t*-test, *t*-statistic −1.31, n.s.; Table 8).

**Table 8:** Combined cartilage and bone repair score results (from ICRS II-2010 and O’Driscoll et al.)

Time period	Group		Nature of predominant tissue		Structural characteristics									Freedom from cellular changes of degeneration		Freedom from degenerative changes in adjacent cartilage	Total performance score	Collagen type II	Collagen type I	Overall assessment
			Tissue morphology	Safranin-O staining of the matrix	Surface regularity	Structural integrity	Thickness	Bonding to the adjacent cartilage	Basal integration	Formation of a tidemark	xSubchondral bone abnormalities/marrow fibrosis	Cancellous bone regeneration	Abnormal calcification/ossification (cartilage tissue)	Hypocellularity	Chondrocyte clustering					
6 months	Agili-C	Mean	3.4	2.4	2.4	1.6	1.9	2.0	4.0	2.6	2.6	2.3	0.0	2.9	0.4	2.6	31.0	3.0	0.4	3.4
		SD	0.9	0.7	0.5	0.5	0.3	0.0	0.0	0.5	1.0	0.7	0.0	0.3	0.5	0.5	4.1	0.0	0.7	0.9
	Empty defect control	Mean	2.0	2.0	1.7	1.0	0.7	1.0	1.3	1.0	0.0	1.3	0.0	2.0	0.7	1.7	16.3	2.0	1.2	2.0
		SD	1.6	0.8	1.2	0.8	0.5	0.8	1.9	1.4	0.0	0.5	0.0	1.4	0.5	1.2	11.8	1.3	1.0	1.6
12 months	Agili-C	Mean	3.7	2.9	2.0	1.7	1.6	2.0	4.0	2.9	2.6	3.6	0.0	2.4	1.0	2.7	33.0	2.9	0.1	3.9
		SD	0.7	0.3	0.8	0.5	0.7	0.0	0.0	0.3	1.0	0.7	0.0	0.7	0.0	0.7	5.5	0.3	0.3	0.3
	Empty defect control	Mean	1.7	2.3	0.3	0.7	1.0	1.7	2.3	1.7	0.0	1.3	0.3	2.0	1.3	1.3	18.0	1.7	0.3	1.0
		SD	0.5	0.9	0.5	0.5	0.8	0.5	1.2	1.2	0.0	0.5	0.5	0.8	0.5	1.2	8.3	1.2	0.5	0.8

*SD* standard deviation

#### 6-month time period

Empty defect control group (*n* = 3)

Two out of three specimens were not healed, and the defects were filled with fibrous tissue and one site healed spontaneously at the cartilage level, with thin layer of hyaline cartilage, but with formation of an irregular subchondral bone with a large bone cavity.

Ar-HA test group (*n* = 7)

Five out of seven sites were healed with formation of hyaline cartilage. In these animals, the formation of hyaline cartilage was confirmed by marked presence of proteoglycans, a marked grade of collagen type II, and absence or traces of collagen type I within this cartilaginous tissue. The weight-bearing surface was quite smooth. The regenerated cartilage was well integrated into the adjacent native cartilage and basal tissues. A newly formed tidemark was observed between the cartilage and the bone tissues. The subchondral plate was largely reconstructed. The underlying cancellous bone was moderately reconstructed.

Two out of seven sites were healed with generation of a fibrocartilage tissue. One of these animals showed delayed subchondral bone reconstruction.

#### 12-month time period

Group A (*n* = 3)

None of the sites had fully healed. Only one of the sites showed advanced signs of cartilage healing, but the subchondral bone formation was not achieved.

There were none to mild signs of inflammation with infiltration of macrophages, giant cells, and fibrin observed in the repair tissue. The neovascularization was of slight grade. One specimen showed incomplete signs of healing with presence of intra-cartilage clefts, remaining active fibroblasts and fibrous tissue, hemorrhage, and delayed subchondral bone plate formation. One specimen displayed cartilaginous repair tissue with morphological evidence of hyaline cartilage as confirmed by the presence of proteoglycans and collagen type II and absence of collagen type I within this cartilaginous tissue. However, this specimen was not considered as fully healed due to a noticeable deformation of the subchondral plate indicating incomplete bone and cartilage repair. The third specimen showed no cartilage formation with a small area of denuded bone.

Group B (*n* = 7)

In group B, six out of seven sites displayed formation of a hyaline cartilage repair tissue. The remaining site was almost fully healed and showed hyaline cartilage formation in most parts (Fig. 2).

Residual signs of local inflammation were observed in only two out of seven sites. No significant signs of neovascularization were observed in any of the specimens. Six out of seven specimens exhibited cartilaginous repair tissue with morphological evidence of hyaline cartilage. The hyaline cartilage features were confirmed by the presence of proteoglycans and collagen type II and absence of collagen type I within the cartilaginous tissue. The cartilaginous repair tissue was well integrated into the adjacent native cartilage and basal tissues in all sites. A reconstructed subchondral bone plate with a clear tidemark was observed in all specimens, at the exception of one specimen that showed a small localized thin fibrocartilage layer, on the boundary of one side of the implantation site. In that last specimen, most part of the cartilage layer was of hyaline structure. The underlying cancellous bone was reconstructed in all specimens in a marked process of osteotransduction. In two specimens out of seven, a bone cavity, filled with adipocyte bone marrow, was observed deep in the bone phase, between the bottom of the implant and the floor of the implantation site.

## Discussion

Current cartilage repair therapies are notorious for their variable outcomes [24, 25]. There is an unmet need for a predictable and reliable therapy.

The current study evaluates up to 1-year performance of the Ar-HA biphasic scaffold implant in a goat model in comparison with a defect in which blood clot extruding from the bone marrow is allowed to form. The control group is similar to a marrow stimulation technique [26], which is widely clinically used. The 6-mm diameter osteochondral defect cannot spontaneously repair in a goat model [15] and thus is of critical size [13, 16]. Indeed, none of group A animals had full cartilage and bone repair.

The goat cartilage repair model is frequently used to study osteochondral grafting and meniscal repair [27, 28], as the knee joints are large enough to create lesions similar in size to those treated in human patients[17].

In contrast to the marrow stimulation induced by the osteochondral defect creation that failed to produce high-quality repair, Ar-HA implants induced high-quality repair tissue formation with long-term durability, similar to previous shorter-term follow-up results [9].

Cartilage is devoid of nerve fibers, and pain generation appears to be subchondral bone related, thus, replacement of the subchondral bone could be advocated in cartilage lesions as abnormal subchondral bone appears to be related to osteoarthritis progression [29]. The advantage of the Ar-HA implant compared to superficial cartilage treatment relates to reconstruction of the osteochondral bone in an osteotransduction process [30]. A concern with osteochondral replacement in chondral lesions might be inconsistent bone regeneration leading to geode formation. In this study, the Ar-HA-based scaffold proved to consistently support osseous and cartilaginous tissue formation lending confidence to osteochondral replacement in full-thickness cartilage lesions.

The chondral phase of the implant stimulates formation of hyaline cartilage due to a composite of modified AR with HA. HA is critical for the maintenance of the physico-chemical characteristics of extracellular cartilage matrix, with both chondrogenesis and chondro-protection effects [31]. HA bonded to a substrate exhibits a size-dependent stimulation of chondrogenic differentiation [32] and influences cell motility, cell differentiation, and cell development. In previous studies [10], the unique spatial orientation of the implant was recognized to regenerate cartilage.

The bone phase of the Ar-HA implant is an aragonite derivative, an osteo-conductive and osteo-transductive nanomaterial, reminiscent of human bone due to its 3D structure and crystalline form of calcium carbonate (CaCO_3_), together with macro- and micro-porosity and pore interconnections [33, 34]. In addition, pore interconnectives are optimal for the development of Haversian systems and the essential entry of blood vessels [35, 36].

An advantage of the current study is the multimodality imaging assessment of the repair tissue. Ultrasound confirmed the superiority of the superficial repair tissue in group B, while micro-CT results indicate that in group B animals, the regenerated bone is similar in density to the native bone, in stark contrast with the group A animals. MRI confirmed the superior repair tissue formation of group B animals. The articular degeneration mitigation of the implant is evidenced by MRI findings (T2 mapping) of surrounding articular cartilage preservation in the implanted group as opposed to cartilage degeneration in the control animals. This corroborates results by Schinhan et al. [13] that such defects in the medial tibiofemoral joint of the goat consistently induce osteoarthritic changes if left untreated. Mesenchymal cell injection into the knee has previously been shown to retard osteoarthritis progression [37], however, this is the first report that a cell-free scaffold can retard osteoarthritic changes in the cartilage surrounding a focal defect.

The histological results in this study are closely related to the MOCART scoring. Consistency of the repair obtained with the scaffold implantation is attested by the very small standard deviation in the treated group imaging and histological results.

A limitation of the study is the limited follow-up. A 1-year follow-up time point is common in animals due to their limited lifespan. However, human studies often require 2-year follow-up as a minimum.

A pre-requisite for extensive use of a novel osteochondral implant is its safety. In the current study, no (SAE) were observed in the Ar-HA group and one non-related SAE occurred in a group A animal. After 6 and 12 months, no significant local inflammation was observed in both groups, thus supporting the biocompatibility and safety of this scaffold.

The Ar-HA scaffold led to restoration of the articular surface without the need of exogenous cells. Clinical application of this technique has to be tested in future studies. The technique might allow simplification of the osteochondral treatment algorithm. An off-the-shelf, single-stage solution for the treatment of cartilage defects is potentially of great importance.

## Conclusions

This study assessed the long-term efficacy and safety of the Ar-HA implant. The scaffold allowed full restoration of both a high-quality cartilage as well as the subchondral bone. The regenerated tissues appear to undergo further maturation and remodeling from the 6- to the 12-month follow-up. These results could support the postulate that the Ar-HA scaffold is suitable for the repair of cartilage and osteochondral lesions in humans [10].
